# *Montrichardia linifera* (Arruda) Schott promotes accelerated wound healing *in vitro*: a promising healing

**DOI:** 10.3389/fphar.2025.1512570

**Published:** 2025-05-20

**Authors:** Aline Costa Bastos, Maurício Ferreira Gomes, W. B. S. Pinheiro, Anderson de Santana Botelho, Raimundo Junior da Rocha Batista, Cristine Bastos do Amarante, Taia Maria Berto Rezende, Yasmin Cunha da Silva, Ananda da Silva Antonio, Henrique Marcelo Gualberto Pereira, Valdir Florêncio da Veiga Júnior, André Salim Khayat, Elizabeth S. Yamada, Gilmara de Nazareth Tavares Bastos

**Affiliations:** ^1^ Laboratory of Neuroinflammation, Institute of Biological Sciences, Federal University of Pará, Belém, Brazil; ^2^ Oncology Research Center, Federal University of Pará, Belém, Brazil; ^3^ Laboratory of Central Extraction, Institute of Exact and Natural Sciences, Federal University of Pará, Belém, Brazil; ^4^ Laboratory of Chemical Analysis, Coordination of Earth Sciences and Ecology, Emílio Goeldi Museum, Belém, Brazil; ^5^ Faculty of Health Sciences, University of Brasilia, Brasilia, Federal, Brazil; ^6^ Chemical Engineering Section, Military Institute of Engineering, Rio deJaneiro, Brazil; ^7^ Laboratory for the Support of Technological Development, Chemistry Institute, Federal University of Rio de Janeiro, Rio deJaneiro, Brazil; ^8^ Institute of Biological Sciences, Federal University of Pará, Belém, Brazil; ^9^ Laboratory of Experimental Neuropathology, Institute of Biological Sciences, Federal University of Pará, Belém, Brazil; ^10^ Laboratory of Developmental Biology, Department of Morphology, Federal University of São Paulo, SãoPaulo, Brazil

**Keywords:** *Montrichardia linifera*, healing, skin injury, natural product, wound

## Abstract

**Introduction:**

*Montrichardia linifera* (Arruda) Schott (*M. linifera*) is commonly used by Amazonian riverine communities for the treatment of skin ulcers, although its effects as a wound healer have never been evaluated until now. Therefore, the *in vitro* wound-healing activity of the extracts from the stem and petiole of *M. linifera* was investigated for the first time.

**Methods:**

The extracts were characterized by chromatography coupled with spectroscopic or spectrometric methods (HPTLC-UV and UHPLC-MS), and free radical scavenging was verified using bioautography with the DPPH radical. Cytotoxicity was evaluated through the MTT method, and a scratch assay was employed to assess cell migration, while *in vitro* cell proliferation was evaluated through immunofluorescence for BrdU-positive cells.

**Results:**

Chemical characterization revealed the presence of 13 metabolites in ESML and EPML extracts. Analytical analysis of the extract demonstrated the elimination of free radicals by autobiography. The extracts did not demonstrate cytotoxicity in fibroblasts and cell migration and proliferation were, significantly, increased reducing the wound area *in vitro*.

**Conclusion:**

Thus, it was observed that the extracts from the stem and petiole of *M. linifera* possess potential wound-healing effects in fibroblasts *in vitro*. This is a pioneering study that provides insights for future studies on the mechanisms of action of this species, in addition to validating the ethnopharmacological knowledge of this species used in the Amazon.

## 1 Introduction

Wounds or skin lesions are characterized by the disruption of the skin, which, depending on the severity, may compromise its structure and function. Following a skin injury, the healing process is initiated to restore tissue homeostasis. Wound healing is divided into three phases: inflammation, proliferation, and remodeling (Gurtner et al., 2008; Strbo et al., 2014).

During the proliferation phase, there is a massive presence of dermal fibroblasts, cells that are closely involved in the healing process. These cells migrate, proliferate, differentiate, and contribute to the restoration of damaged tissue through a series of crucial actions for tissue repair, such as the release of growth factors, protein synthesis, and tissue contraction (Bainbridge, 2013).

Dysregulation of this phase of wound healing impairs the progression of the tissue repair process and worsens the injury leading to a chronic condition (Demidova-Rice et al., 2012). Chronic wounds can occur throughout an individual’s lifetime; however, people with comorbidities and the elderly are more susceptible to these lesions (Serra et al., 2018; El Mohtadi et al., 2021). The presence and severity of the lesions can cause complications for patients, such as increased morbidity, low self-esteem, infections, amputation, and, in some cases, death (Salomé et al., 2016; Cefalu et al., 2017; Kim et al., 2011; Walsh et al., 2016).

Methods are used to prevent and treat skin lesions, such as repositioning bedridden patients, wound asepsis, the use of anti-inflammatory agents, techniques like debridement, and the application of dressings and local healing agents (Eriksson et al., 2022). For the healing of chronic wounds, positive modulation of healing events is necessary; however, the treatment of these wounds involves high financial costs and does not always produce the desired outcome. Therefore, the discovery of new healing agents with greater efficacy and lower cost is necessary (Olsson et al., 2019; Lindholm and Searle, 2016; Nussbaum et al., 2018).

**FIGURE 1:**
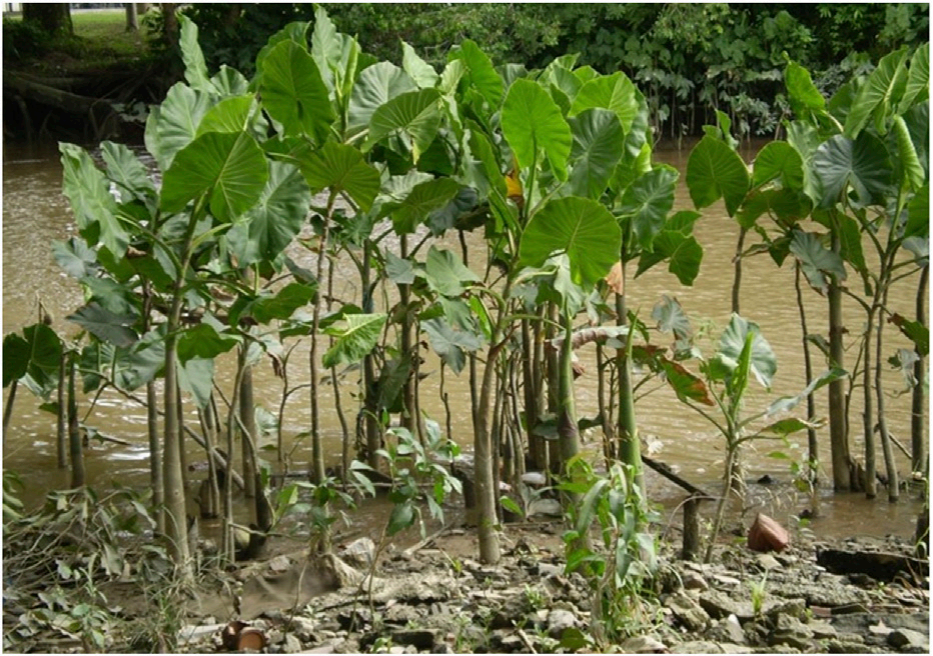
*M. linifera*. Photograph of the botanical species *M. linifera* in its natural environment. The M. linifera is predominantly found in floodplain areas, as shown in the photograph.

Given this, natural products are used in various regions of the world as therapeutic agents for skin wounds (Albahri et al., 2023; Shedoeva et al., 2019), the *Montrichardia linifera* (Arruda) Schott (*M. linifera*) is one example (Figure 1). This species belongs to the Araceae family, and is commonly found in the Amazon region, and studies confirming its pharmacological activity are scarce. However, some activities of *M. linifera* have already been described, such as antiplasmodial, anti-leishmanial, antibacterial, and antioxidant properties (Amarante et al., 2011; Costa et al., 2009; Faria et al., 2021; Lima et al., 2021; Miranda et al., 2015; Santos et al., 2014). It was also reported by Plowman (1969) the use of this plant for the home treatment of skin wounds and as an anti-rheumatic by Amazonian communities (Plowman, 1969). Despite its ethnopharmacological use, the wound healing activity of *M. linifera* has not yet been proven. Therefore, the aim of this study was to investigate the wound healing activity of the stem extract of *M. linifera* (ESML) and the petiole extract of *M. linifera* (EPML) in a fibroblast cell line.

## 2 Materials and methods

### 2.1 Collection and extraction of the plant material

The petiole and stem samples of *M. linifera* were collected in the aningal of the Mangal das Garças, along the banks of the Guajará Bay/Guamá River (coordinates: S 1.46471° and W 48.50674°), located in the city of Belém do Pará. The specimen is registered with the National System for the Management of Genetic Heritage and Associated Traditional Knowledge (SisGen - Brazil) under registration number A91B68B, and a specimen is deposited in the João Murça Pires Herbarium of the Emílio Goeldi Museum (MG 216695).

After collection, the samples of *M.linifera* petiole and stem were washed in running water. Then, they were dried in an air-circulating oven at a temperature of 60°C for a period of 24 h, and after drying, they were ground in a knife mil. A mass of 100 g of petiole and stem powder was subjected to maceration extraction with ethanol as a solvent. After a 48 h extraction period, the solutions were filtered through qualitative filter paper and concentrated using a rotary evaporator Model Q344M2, with a thermostatic bath Model Q214M2, brand QUIMIS^®^. Subsequently, they were dried in an oven with air circulation at 45°C to obtain the dry extracts. The resulting crude extracts were weighed and stored in a freezer at −18°C.

### 2.2 Characterization by high-performance thin-layer chromatography (HPTLC)

The chemical profile was performed using a robotic system comprising the application modules (Automatic TLC Sample 4 – ATS4) and the TLC Visualizer photodocumentation system (CAMAG - Muttenz, Switzerland). Silica gel aluminum plates F-254 60 Å from SILICYCLE (Quebec, Canada) were used as the stationary phase, and HPLC-grade solvents (TEDIA^®^ COMPANY–Fairfield, USA) were used as the mobile phase.

Aliquots of 50 µg of the extracts and 1 µg of each metabolite standard were applied to the chromatoplates and eluted in a glass chamber using an isocratic system of dichloromethane/methanol/formic acid (94:5:1) to assess the presence of terpenes/steroids and dichloromethane/methanol/water/formic acid (77:20:2:1) to evaluate the presence of saponins, phenolic compounds, flavonoids, and alkaloids. Subsequently, the chromatoplates were derivatized with selective developing solutions for the evaluated metabolite classes, prepared according to the methodology described by Wagner and Bladt (2001), photodocumented, and identified by comparison with the coloration of the standards used against the developing solutions. Image analysis was performed using WinCats 1.4.6 software.

The evaluation of the free radical scavenging capacity of the metabolites presents in EPML and ESML through bioautography was performed by HPTLC, using the same parameters described in the previous test and the elution system dichloromethane/methanol/water/formic acid (77:20:2:1). After elution, the chromatoplate was sprayed with a 40 mM DPPH• radical solution in methanol, stored for 60 min protected from light and oxygen, and photodocumented under visible light. Image analysis was performed using WinCats 1.4.6 software.

### 2.3 Characterization by reversed phase ultra-high performance liquid chromatography coupled with high resolution mass spectrometry (RP-UHPLC-HRMS)

The RP-UHPLC-HRMS analysis were performed with the samples prepared at a concentration of 1 mg/mL in HPLC-grade methanol and filtered through a 13 mm PTFE filter with a pore size of 0.45 µm. The extracts ESML, and EPML were analyzed using a Dionex Ultimate 3000 UHPLC chromatograph (Thermo Scientific, Bremen, Germany) coupled to a Q-Exactive high-resolution spectrometer (Thermo Scientific, Bremen, Germany). A reversed phase C18 column (Syncronis, 2.1 × 50 mm, 100 Å–Thermofisher Scientific, Waltham, United States) was used as the stationary phase, which was maintained at 40°C. Solvent A was formed by 0.1% formic acid in deionized water: 5 mM ammonium formate and solvent B was formed by methanol acidified with 0.1% formic acid. The gradient started with 5% B up to 0.3 min, 10% B up to 0.5 min, 25% B up to 1 min, 90% B up to 6 min, 100% B up to 8 min, 100% B up to 9 min, 5% B up to 9.1 min and 5% B up to 11.1 min. A flow rate of 0.4 mL min-1 was used. Mass spectra were obtained in full scan and ms^2^ by data dependent acquisition mode, in positive and negative ionization modes, with a detection range of 150–800 m/z. Data processing was performed using Xcalibur 3.0.1 software.

### 2.4 Cell culture and cellular cytotoxicity analysis

The fibroblast cell line L929 (ATCC) was cultured in high-glucose DMEM supplemented with 10% fetal bovine serum (FBS) (Gibco^®^), 10 mM HEPES (Sigma), 2 mM L-glutamine, 1% MEM non-essential amino acids (Sigma), 100 U/mL streptomycin and 100 U/mL penicillin (Thermo Fisher), and 0.2 μg/mL amphotericin B (Vitrocell).

The fibroblasts were seeded at 2.5 × 10^3^ cells/well in 96 well plates (KASVI), with a total volume of 100 μL, at 24 h in a humidified incubator at 37°C with 5% CO₂. Subsequently, the ESML and EPML extracts were solubilized in DMSO and then diluted immediately before treatment in media without FBS, ensuring that the DMSO concentration did not exceed 0.01% in each treated group. In the wells with the cell monolayer, treatment with media without FBS, or with EPML or ESML, at serial concentrations (100, 50, 25, 12.5, 6.25, 3.125, 1.56, 0.78, 0.39, 0.19 μg/mL) of each extract for up to 24, 48, and 72 h. The treatment solutions were added without removing the previous medium (with 10% FBS), then culture medium in the experimental groups during the assay contained a low concentration of FBS (5%).

Since the extracts were solubilized in DMSO, a preliminary MTT assay was performed using a concentration curve of this solvent (ranging from 1% to 0.001%) in L929 cells to determine the highest non-cytotoxic concentration that could be used in the treatment solution. The solvent was diluted in medium without SBF. This approach aimed to minimize variability related to DMSO usage.

After these time points, the medium was aspirated, and 0.5 mg/mL of MTT (Sigma) was added to the cells and incubated for 3 h at 37°C. Following incubation, the MTT solution was removed, 100 µL of DMSO (Sigma) was added to solubilize the formazan crystals, and the plate was read using a digital microplate reader, model Polaris - Celer Biotecnologia S.A. (λ = 570 nm). The control group was considered to have 100% viability. The experiment was performed in triplicate (n = 8), in three independent experiments.

### 2.5 Cell migration assessment

The cells were seeded at a density of 2.9 × 10⁴ cells/well in 12 well plates and incubated overnight, showing 70%–80% cell confluence. A scratch was then made in the center of each well using a sterile 10 µL pipette tip, creating the scratch at a 90° angle between the pipette tip and the plate, followed by a wash to remove the detached cells. The cells were then treated with media without FBS or ESML or EPML extracts solubilized in DMSO. This solution (extract and DMSO) was diluted, immediately before treating the cells, ensuring that the DMSO concentration did not exceed 0.01% in each treated group, with media without FBS to achieve concentrations of 0.78, 0.39, and 0.19 μg/mL. The treatment solutions were added without removing the previous medium (with 10% FBS), then culture medium in the experimental groups during the assay contained a low concentration of FBS (5%). Microphotographs of all wells were taken for up to 0, 6, 12, and 24 h using the Stereologer^®^ software (Stereology Resource Center, Inc., version 11.0) connected to the Medilux TCM400 inverted microscope bright-field. The wounded area in the fibroblast monolayer was quantified using ImageJ^®^ software.

To quantify the pixel area of the microphotographs from the cell migration assay, the images were opened in the ImageJ 1.52a software and converted to 8-bit format. Then, the FFT plugin was used, where the ‘Bandpass Filter’ dialog box was opened, and the options ‘Autoscale after filtering’ and ‘Saturate image when autoscaling’ were selected. Next, the ‘Threshold’ dialog box was opened, and the ‘Dark background’ option was selected. A minimum filter was then applied and standardized to a radius of seven pixels. After applying the filters, the wound area was selected using the magic wand tool and measured.

The pixel areas were normalized and converted into percentages, with the 0 h time point considered as 100% of the wounded area. To better visualize the morphology and to delimit the injury area, hematoxylin and eosin staining was performed at the 24 h time point. For this, after 24 h of treatment, the wells containing the cells were washed twice with phosphate-buffered saline (PBS) and then fixed with 4% paraformaldehyde at 37°C for 10 min under agitation. Subsequently, the wells were washed with PBS and stained with hematoxylin for 7 min under agitation. After another wash with PBS, eosin was added to the wells for 15 min under agitation. Following this period, a final wash was performed, and the wells were microphotographed. The experiment was performed in triplicate (n = 3), in three independent experiments.

### 2.6 Cell proliferation assessment

The cells were seeded at a density of 8 × 10⁴ cells/well in 24 well plates and cultured overnight on coverslips pre-treated with poly-L-lysine Subsequently, the cells were then treated with media without FBS or ESML, or EPML extracts solubilized in DMSO. This solution (extract and DMSO) was diluted, immediately before treating the cells, ensuring that the DMSO concentration did not exceed 0.01% in each treated group, with media to achieve concentrations of 0.78, 0.39, and 0.19 μg/mL applied for 24 h. The BrdU marker (5-bromo-2-deoxyuridine) at 10 µM was added 2 h before fixation. The cells were stained with anti-BrdU (1:1000) (Sigma Aldrich^®^) for 24 h at 4°C and then incubated overnight with Cy3 donkey anti-mouse antibody (1:500) (Jackson Immuno Research Laboratories, Inc.) for 2 h. The cells were counterstained with DAPI(1:100,000) for 20 min. Microphotographs were taken using the Axio Imager.M2 fluorescent microscope (ZEISS). The number of BrdU-positive cells was quantified using a multi-point tool of ImageJ^®^ 1.52a software. The experiment was performed in triplicate (n = 3), in three independent experiments.

### 2.7 Statistical analysis

Statistical evaluation was conducted using the One-Way ANOVA test with Bonferroni post-test for the cytotoxicity assay; the Two-Way ANOVA test with Tukey post-test for migration analysis; and the One-Way ANOVA with Tukey post-test for cell proliferation assessment. All statistical analyses were performed using GraphPad Prism six software, version 6.01. A significance level of p < 0.05 was considered for the applied tests.

## 3 Results

### 3.1 Analysis of ESML and EPML extracts by HPTLC

**FIGURE 2:**
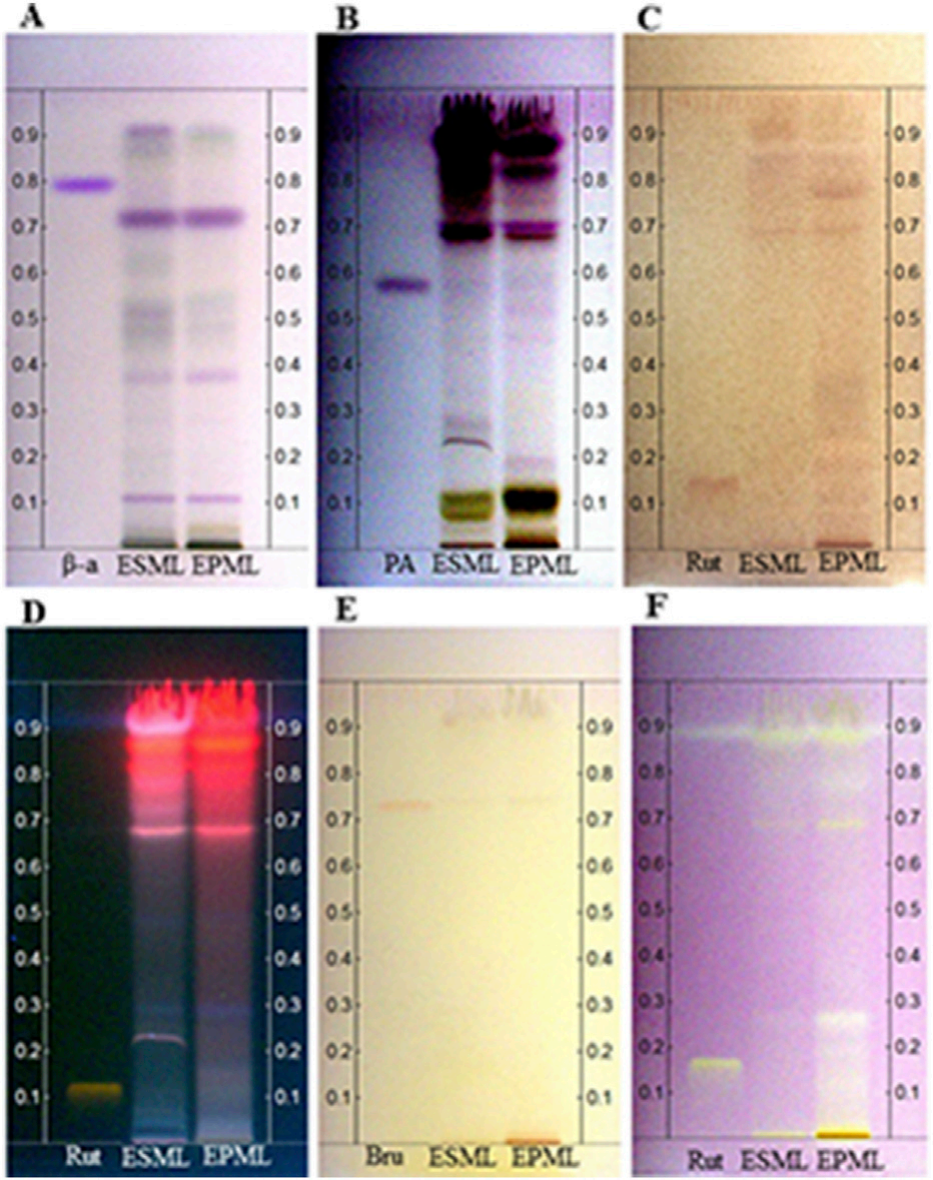
Chromatoplates developed for the analysis of the chemical profile by HPTLC of ESML, and EPML. Through HPTLC, the presence of substance classes such as terpenes/steroids, saponins, phenolic compounds, flavonoids, and alkaloids was indicated in the ESML, and EPML extracts. **(A)** Purple bands corresponding to terpenes/steroids were revealed using VAS solution, and **(B)** Purple bands corresponding to saponins were revealed with VAS solution, **(C)** Reddish-brown streaks after spraying with FBS, **(D)** Chromatographic plate for the identification of bands corresponding to flavonoids using NP/PEG developers, **(E)** Chromatographic plate for identification of alkaloid bands using Dragendorff developer, **(F)** Yellow bands corresponding to free radical neutralization of EPML and ESML. β-amyrin (β-a); Pulsatilla A (PA), Rutin (Rut), Brucin (Bru), ESML (Stem) and EPML (Petiole).

The analyses performed by HPTLC allowed for the characterization of EPML and ESML regarding the presence of several important secondary metabolites. In the chromatogram presented in Figure 2A, it was possible to identify the presence of terpenes/steroids in both extracts, characterized by the formation of purple bands at retention factors (Rf) of 0.10, 0.38, and 0.72 after spraying with VAS solution (1% (m/v) ethanolic solution of vanillin and 10% sulfuric acid), as observed in the β-amyrin (β-a) standard, as well as the similarity between the extracts in their terpene/steroid composition.

In the chromatogram shown in Figure 2B, the presence of saponins in the extracts was identified, characterized by the formation of faint purple bands at Rf values of 0.27 for ESML, and 0.19, 0.47, and 0.51 for the petiole extract after spraying with VAS solution, as observed in the Pulsatilla A. (PA) standard. Additionally, the presence of phenolic compounds in the extracts was also identified (Figure 2C), characterized by the formation of reddish-brown bands after spraying with a 0.5% (m/v) aqueous solution of Fast Blue Salt (FBS), followed by a 10% (m/v) ethanolic sodium hydroxide solution, at Rf values of 0.70, and 0.90 for the stem extract and Rf values of 0.05, 0.11, 0.18, 0.70, 0.78, and 0.90 for the petiole extract, as observed for the Rutin (Rut) standard.

It is also noteworthy, based on the intensity of the formed bands, that the petiole extract exhibits a higher presence of phenolics than the stem extract. The methods used did not allow for the identification of flavonoids and alkaloids in either extract (Figures 2D, E). Based on these results, three lower concentrations of each extract were selected for further assays.

The analysis of free radical scavenging of EPML and ESML was also conducted through bioautography using the DPPH• radical, characterized by the formation of yellow bands corresponding to the neutralization of the radical at Rf values of 0.70 and 0.90 for EPML and at Rf values of 0.27, 0.70, 0.78, and 0.90 for ESML (Figure 2F), as observed for the Rut standard (Figure 2C).

The DPPH radical assay is a test for analyzing the phytochemical profile of extracts; however, it does not reflect the interactions of ESML and EPML with biological systems. For a more comprehensive understanding, additional and broader assays in in vitro and/or *in vivo* models are necessary.

### 3.2 RP-UHPLC-HRMS analysis of EPML and ESML

**FIGURE 3:**
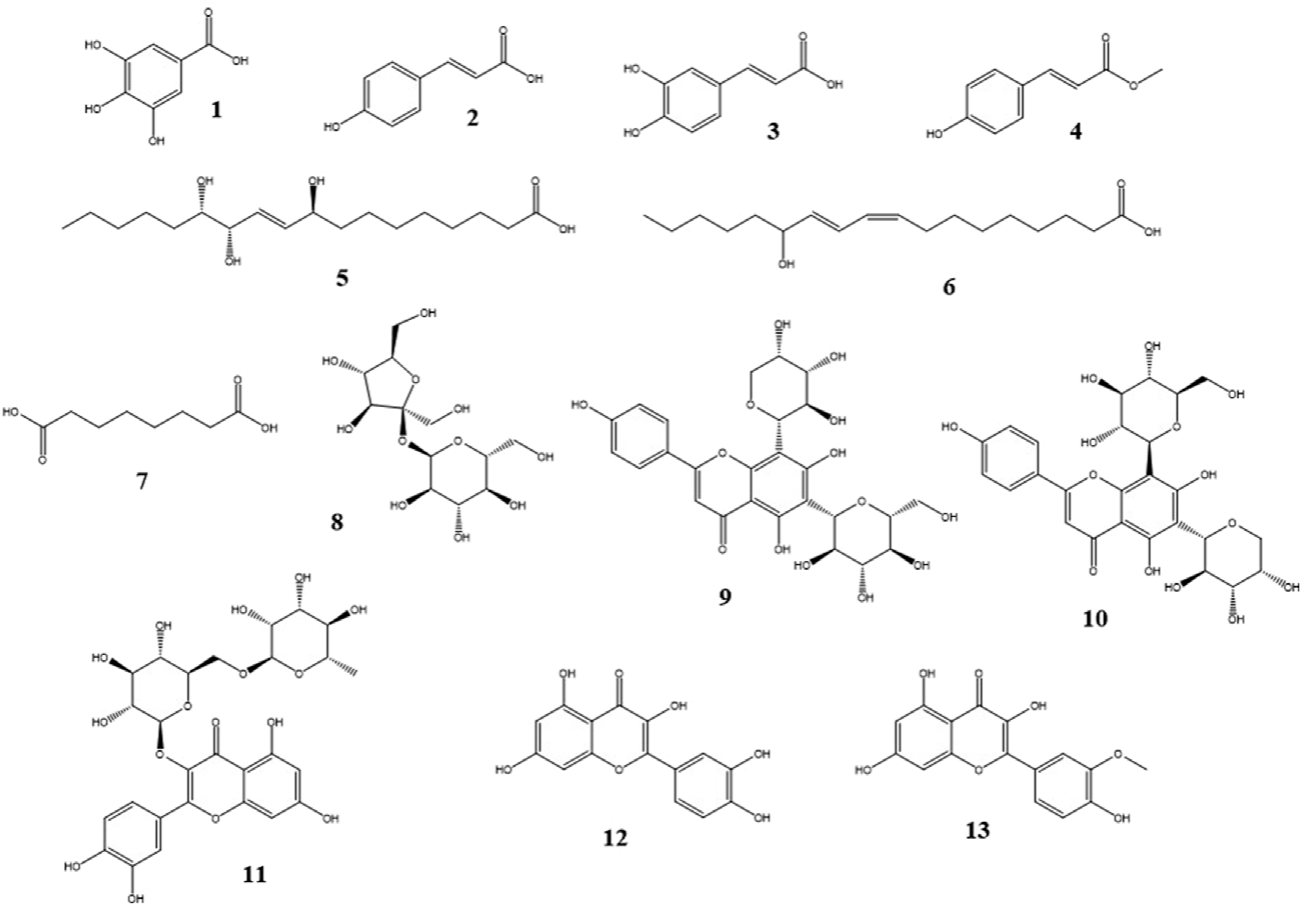
Chemical structures of the metabolites detected in the ESML, and EPML extracts. Thirteen metabolites were detected, with five metabolites common to both extracts. 1) gallic acid, 2) p-coumaric acid, 3) caffeic acid, 4) 4-hydroxycinnamic acid methyl ester, 5) Pinellic acid, 6) coriolic acid, 7) suberic acid, 8) sucrose, 9) scaftoside, 10) isoschaftoside, 11) rutin, 12) quercetin and 13) isorhamnetin.

The samples EPML and ESML were analyzed by RP-UHPLC-HRMS allowing the detection of several substances. Exact mass and fragmentation data were compared with previous published information from the Araceae family. Combining the RP-UHPLC retention times information at the chromatograms and mass spectrometry data, peaks corresponding to sugars, fatty acids, phenolic acids, flavonoids, and anthocyanins were annotated and putatively identified in the extracts ESML, and EPML. Among the substances detected in ESML, the most abundant components were gallic acid, pinellic acid, suberic acid, coriolic acid, p-coumaric acid, and the isomers schaftoside or isoschaftoside (Figure 3). EPML was characterized by a higher number of detected ions. In EPML, the most abundant detected substances were gallic acid, caffeic acid, p-coumaric acid, 4-hydroxycinnamic acid methyl ester, pinellic acid, coriolic acid, suberic acid, sucrose, quercetin, isorhamnetin, and rutin (Figure 3). Additionally, isomers with the same exact mass as the m/z 447.09402 ion, which could correspond to structures such as quercitrin, orientin, isoorientin, luteolin-7-glucoside, kaempferol 3-O-glucoside, apigenin O-pentoside, or cyanidin 3-glucoside, were detected. Other identified isomers included m/z 477.10419 ions corresponding to isorhamnetin 3-galactoside or petunidin 3-O-glucoside; m/z 563.14142 ions corresponding to schaftoside ou isoschaftoside; and m/z 609.14679 ions corresponding to delphinidin 3-rutinoside or cyanidin 3-gentiobioside.

The structures detected in *M.linifera* that have previously been reported within the Araceae botanical family include p-coumaric acid, pinellic acid, schaftoside, isoschaftoside, 4-hydroxycinnamic acid methyl ester, caffeic acid, quercetin, isorhamnetin, quercitrin, orientin, isoorientin, luteolin-7-glucoside, kaempferol 3-O-glucoside, apigenin O-pentoside, cyanidin 3-glucoside, isorhamnetin 3-galactoside, petunidin 3-O-glucoside, delphinidin 3-rutinoside, cyanidin 3-gentiobioside, and rutin (Williams, 1981; Nagai et al., 2002; Su et al., 2013; Wu et al., 2009; Fawzi Mahomoodally et al., 2023; Rechek et al., 2023).

### 3.3 Analysis of cell cytotoxicity caused by EPML, and ESML

The exposure of fibroblast cells to the ESML and EPML extracts induced an increase in viability, and did not promote cellular cytotoxicity at low concentrations.

It was demonstrated that the highest concentration of DMSO in a treatment solution for L929 cells that does not affect cell viability for up to 72 h is 0.01% (Supplementary Figure S1). Therefore, all treatment solutions used in the assays conducted in this study contained a maximum of 0.01% DMSO.

**FIGURE 4:**
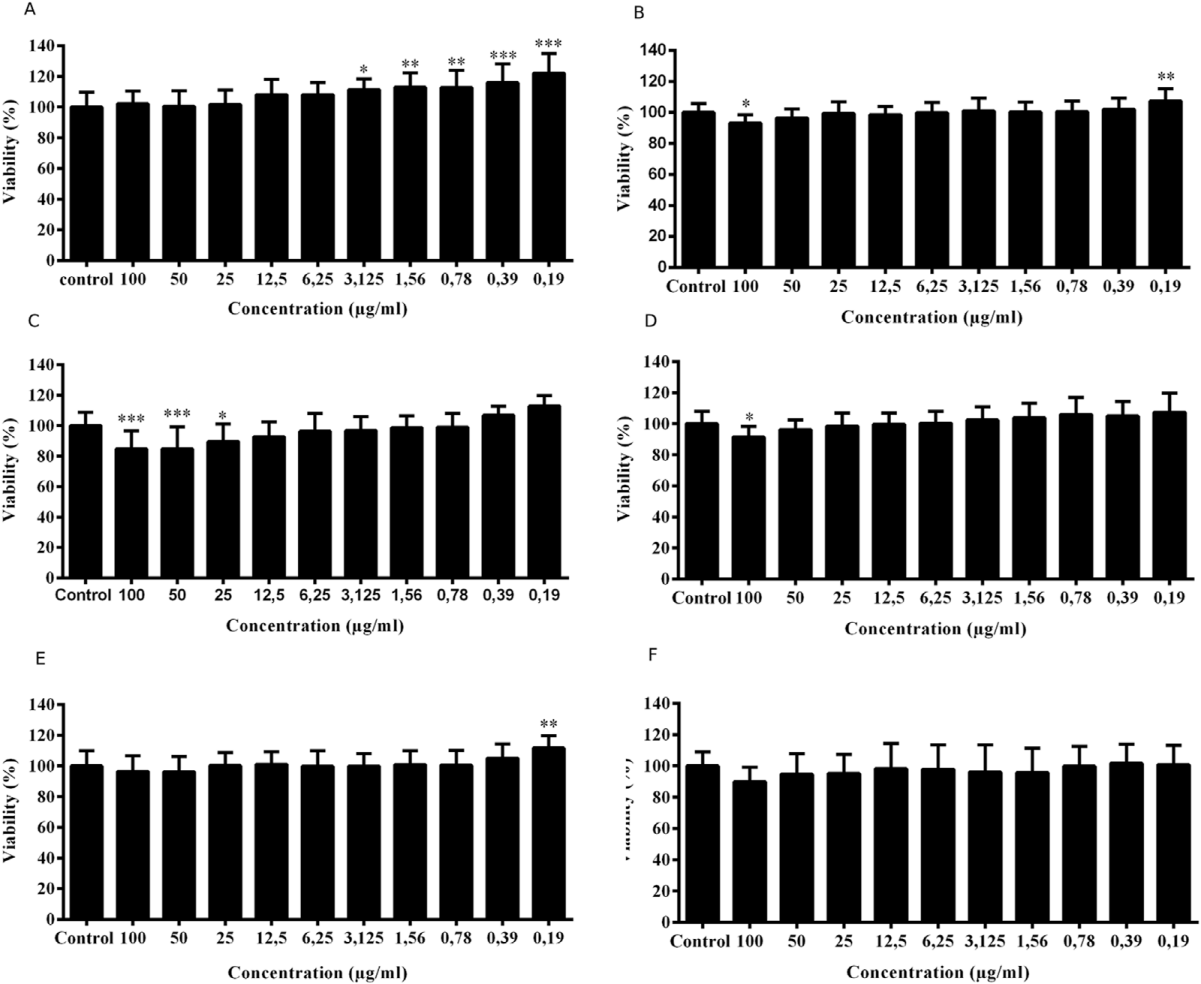
Analysis of the cytotoxic effect of ESML, and ECML extracts on fibroblasts. The viability of L929 cells after exposure to ESML at **(A)** 24 h, **(B)** 48 h, and **(C)** 72 h, and also the exposure to EPML at **(D)** 24 h, **(E)** 48 h, **(F)** 72 h. Significance was considered at *P < 0.05, **P < 0.01, ***P < 0.001 vs control. All groups were used n = 8, performed in triplicate. Mean ± standard deviation.

The cytotoxicity results demonstrated that after 24 h of treatment with EPML, there was a reduction in viability only at the highest concentration used (100 μg/mL) compared to the control group (91.39% ± 6.11%) (Figure 4D). The other concentrations of EPML and all concentrations of ESML did not show a loss of cell viability (Figures 4A, D).

At concentrations of 3.125, 1.56, 0.78, 0.39, and 0.19 μg/mL of ESML, there was a significant increase in cell viability compared to the control, with viabilities of 100.40% ± 8.54%, 102.4% ± 9.19%, 103.90% ± 10.76%, 106% ± 9.51%, and 104.90% ± 12.22%, respectively (Figure 4A).

At the 48, and 72 h time points, there was no decrease in cell viability at any concentration of ESML and EPML used (Figures 4B, C, E, F) compared to control. At the 48 h time point, there was a significant increase in viability at the concentration of 0.19 μg/mL for both ESML and EPML compared to control, with viabilities of 107.40% ± 7.86%, and 111.70% ± 8.01%, respectively (Figures 4B, E). At the 72 h time point, a significant increase was observed at the concentration of 0.19 μg/mL of ESML compared to the control, with viability of 113.6% ± 6.05% (Figure 4C).

### 3.4 Increase in cell migration induced by EPML and ESML

The scratch assay demonstrated an increase in cell migration, indicated by a significant reduction in the area of the lesion created in the cell monolayer when treated with ESML and EPML extracts. This wound closure-inducing activity suggests that these extracts may be enhancing cellular motility.

**FIGURE 5:**
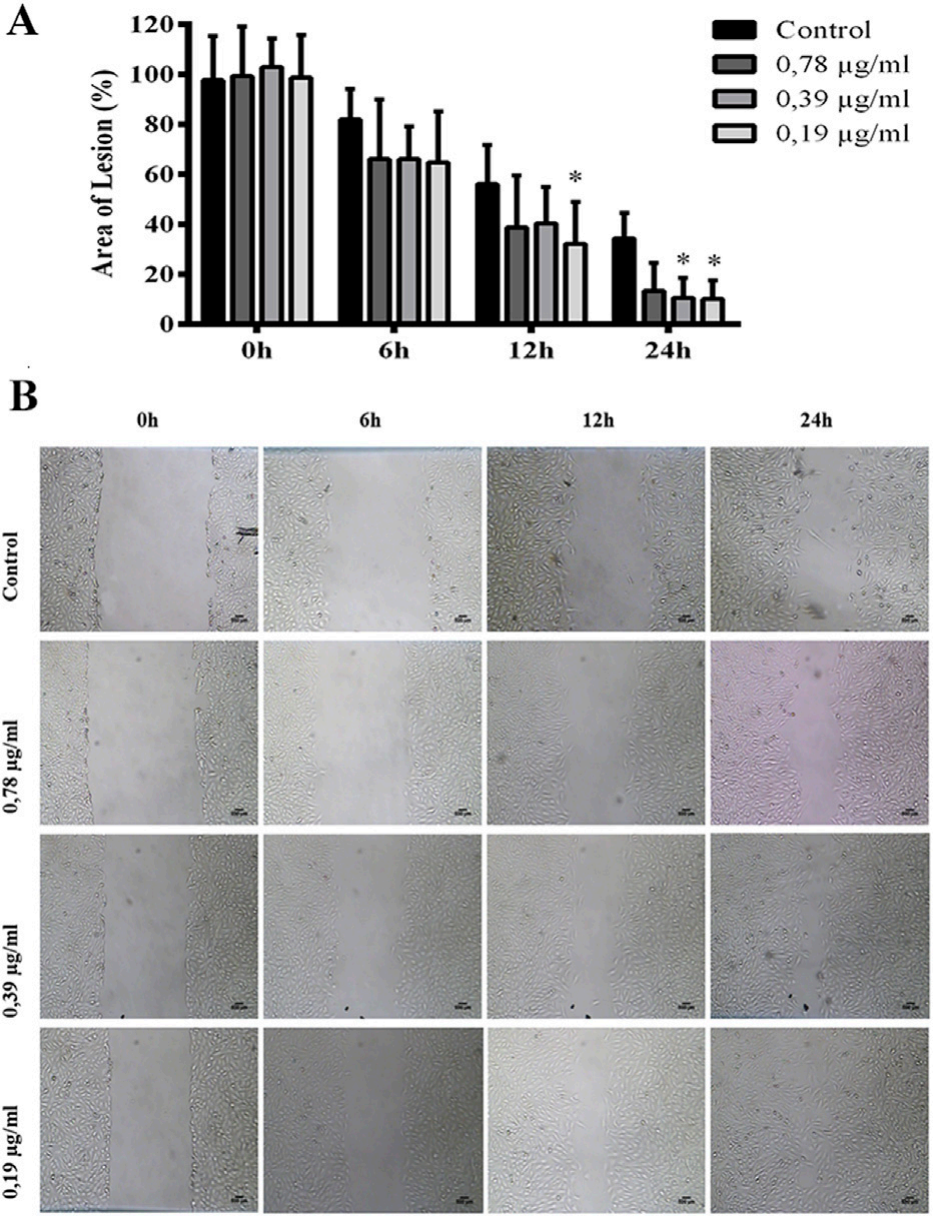
Increased *in vitro* wound healing induced by the ESML extract, analyzed through the scratch assay. Injury area in fibroblast monolayers of the control group, and the group treated with ESML at concentrations of 0.78 μg/mL, 0.39 μg/mL, and 0.19 μg/mL at 0h, 6h, 12h, and 24 h. **(A)** Quantitative data **(B)** Qualitative data. Significance was considered at *P < 0.05, **P < 0.01, ***P < 0.001 vs control. It was performed in triplicate. Mean ± standard deviation. Scale bar in microphotography = 500 µm.

The lesions in the cell monolayer treated with ESML extract at a concentration of 0.19 μg/mL at 12, and 24 h, with lesion areas of 32.29% ± 16.62%, and 10.15% ± 7.35%, respectively. Additionally, there was a significant reduction in the lesion area when treated with the concentration of 0.39 μg/mL of ESML at the 24 h time point, showing a lesion area of 10.67% ± 7.94% (Figure 5).

The 0.78 μg/mL concentration of the ESML extract did not accelerate wound closure *in vitro* compared to the control group at the 6 h, 12 h, and 24 h treatment time. Furthermore, the ESML extract at a concentration of 0.39 μg/mL also did not accelerate wound closure *in vitro* compared to the control group at the 6 h, and 12 h treatment times. Treatment with ESML at a concentration of 0.19 μg/mL for 6 h did not accelerate wound closure in the cell monolayer.

**FIGURE 6:**
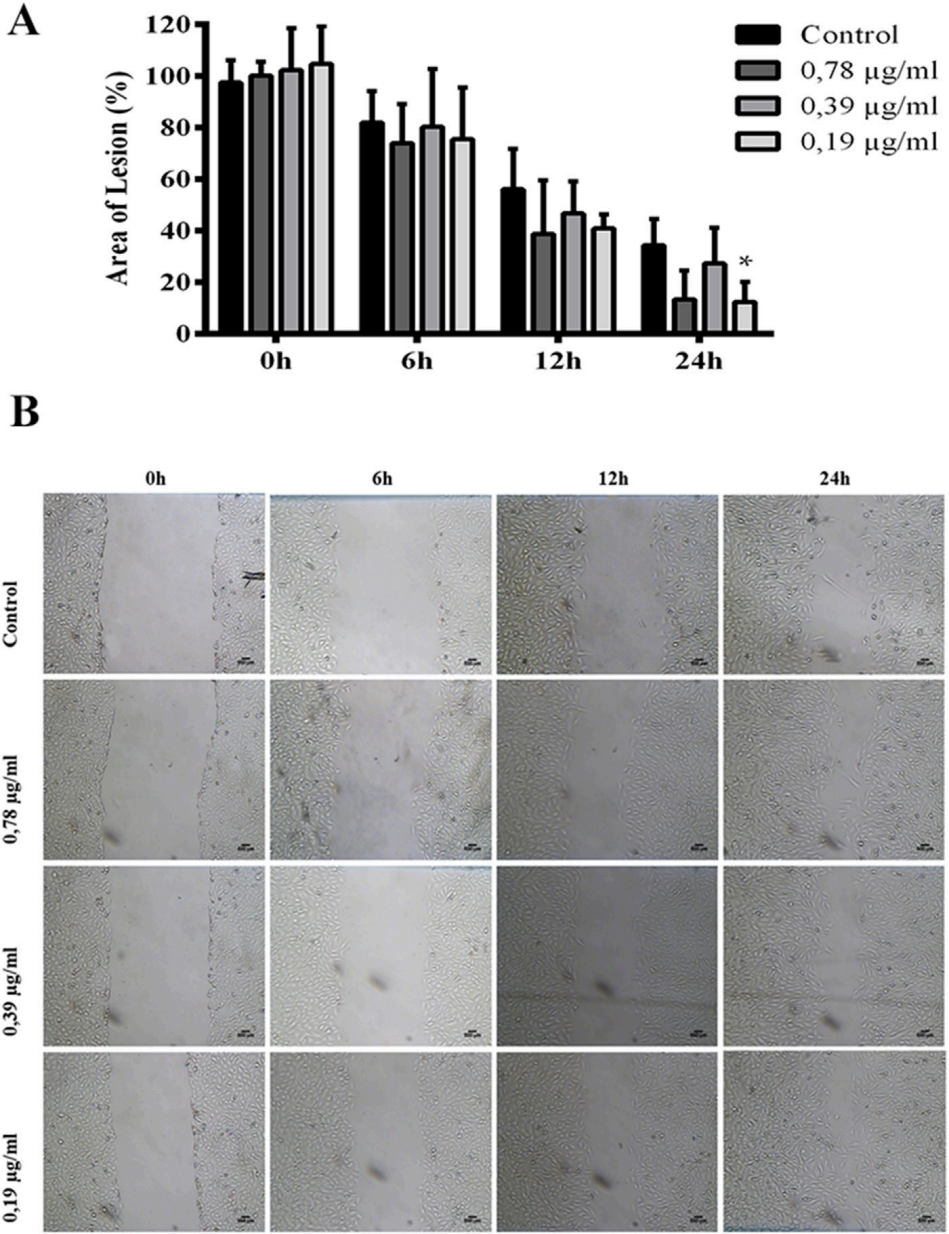
Increased *in vitro* wound healing induced by the EPML extract, analyzed through the scratch assay. Injury area in fibroblast monolayers of the control group, and the group treated with EPML at concentrations of 0.78 μg/mL, 0.39 μg/mL, and 0.19 μg/mL at 0h, 6h, 12h, and 24 h. **(A)** Quantitative data, **(B)** Qualitative data. Significance was considered at *P < 0.05, **P < 0.01, ***P < 0.001 vs control. It was performed in triplicate. Mean ± standard deviation. Scale bar in microphotography = 500 µm.

The treatment of the lesion in the fibroblast monolayer with 0.19 μg/mL EMPL for 24 h demonstrated a significant reduction in the injured area, with an area of 12.40% ± 7.72% (Figure 6).

The concentrations of 0.78 μg/mL, 0.38 μg/mL, and 0.19 μg/mL of the EMPL extract showed no significant difference in wound closure *in vitro* compared to the control at the 6 h, and 12 h time. At 24 h time, the 0.78 μg/mL, and 0.38 μg/mL concentrations of the EMPL extract also showed no significant difference in wound closure *in vitro* compared to the control group.

**FIGURE 7:**
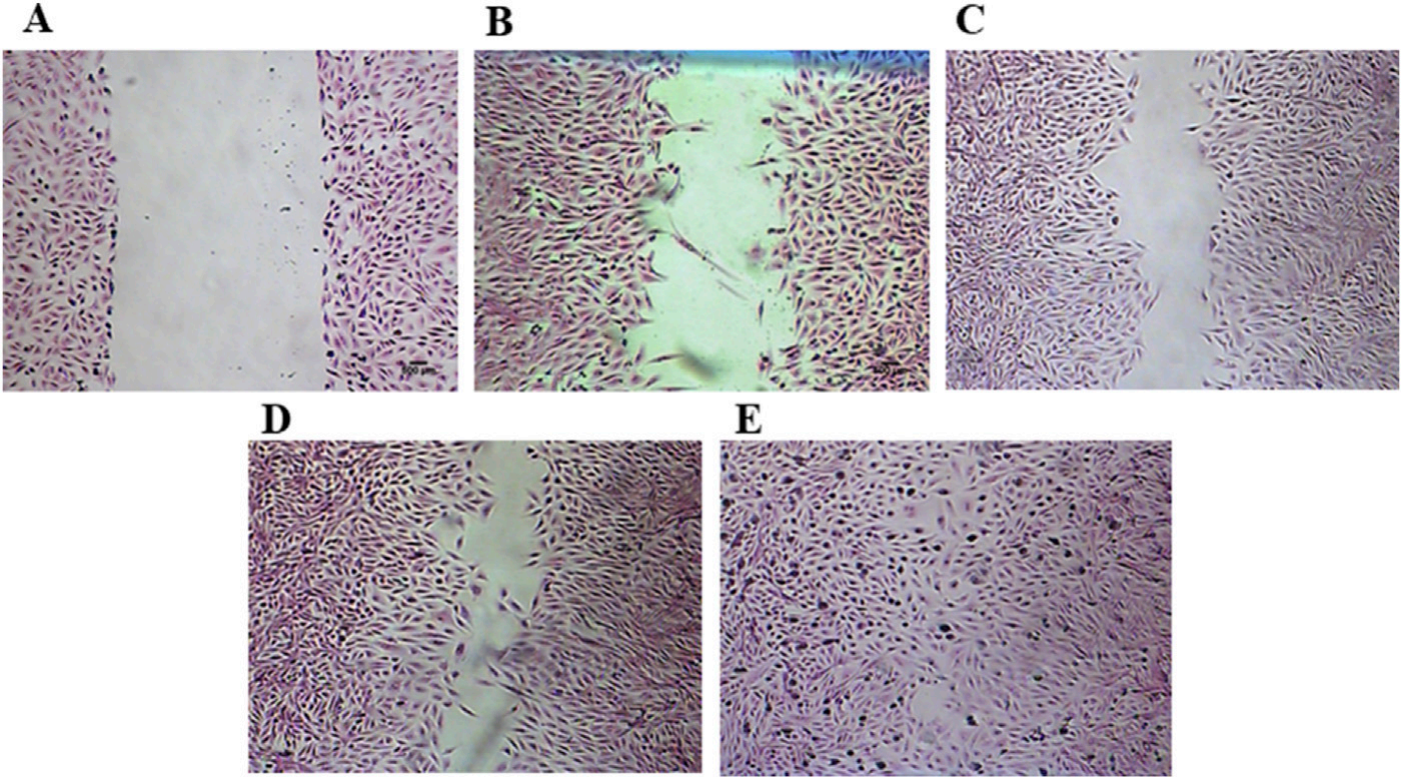
Cell monolayer wound area stained with hematoxylin treated with the ESML extract Injury area in fibroblast monolayers stained with hematoxylin and eosin, treated with ESML. **(A)** control at 0 h, **(B)** control at 24 h, **(C)** 0.78 μg/mL of ESML at 24 h, **(D)** 0.39 μg/mL of ESML at 24 h, **(E)** 0.19 μg/mL of ESML at 24 h. Scale bar in microphotograph = 500 µm.

**FIGURE 8:**
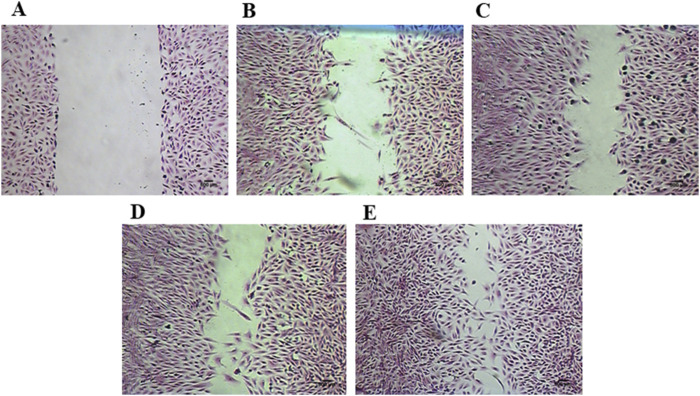
Injury area in fibroblast monolayers stained with hematoxylin and eosin, treated with EPML. **(A)** control at 0 h, **(B)** control at 24 h, **(C)** 0.78 μg/mL of EPML at 24 h, **(D)** 0.39 μg/mL of EPML at 24 h, **(E)** 0.19 μg/mL of EPML at 24 h. Scale bar in microphotograph = 500 µm.

In the Hematoxylin and Eosin staining, it is possible to observe a decrease in the lesion area, attributed to the increased migration of fibroblasts induced by treatment with 0.19, and 0.39 μg/mL of ESML for 24 h (Figures 7D, E), and 0.19 μg/mL of EPML for 24 h (Figures 8D, E).

The *in vitro* lesions were stained to enhance visualization of the wound areas treated for 24 h with ESML, and EPML extracts.

### 3.5 Increase in cell proliferation induced by EPML and ESML

The extracts demonstrated wound closure *in vitro*, raising the hypothesis that, in addition to the ESML and EPML extracts inducing increased cell migration, they might also accelerate fibroblast proliferation. To investigate this, a cell proliferation assay was performed.

The proliferation rate induced by ESML and EPML was analyzed through BrdU immunostaining, marking the cells that entered the S phase of the cell cycle. The control group showed 19.78 ± 3.80 BrdU positive cells.

**FIGURE 9:**
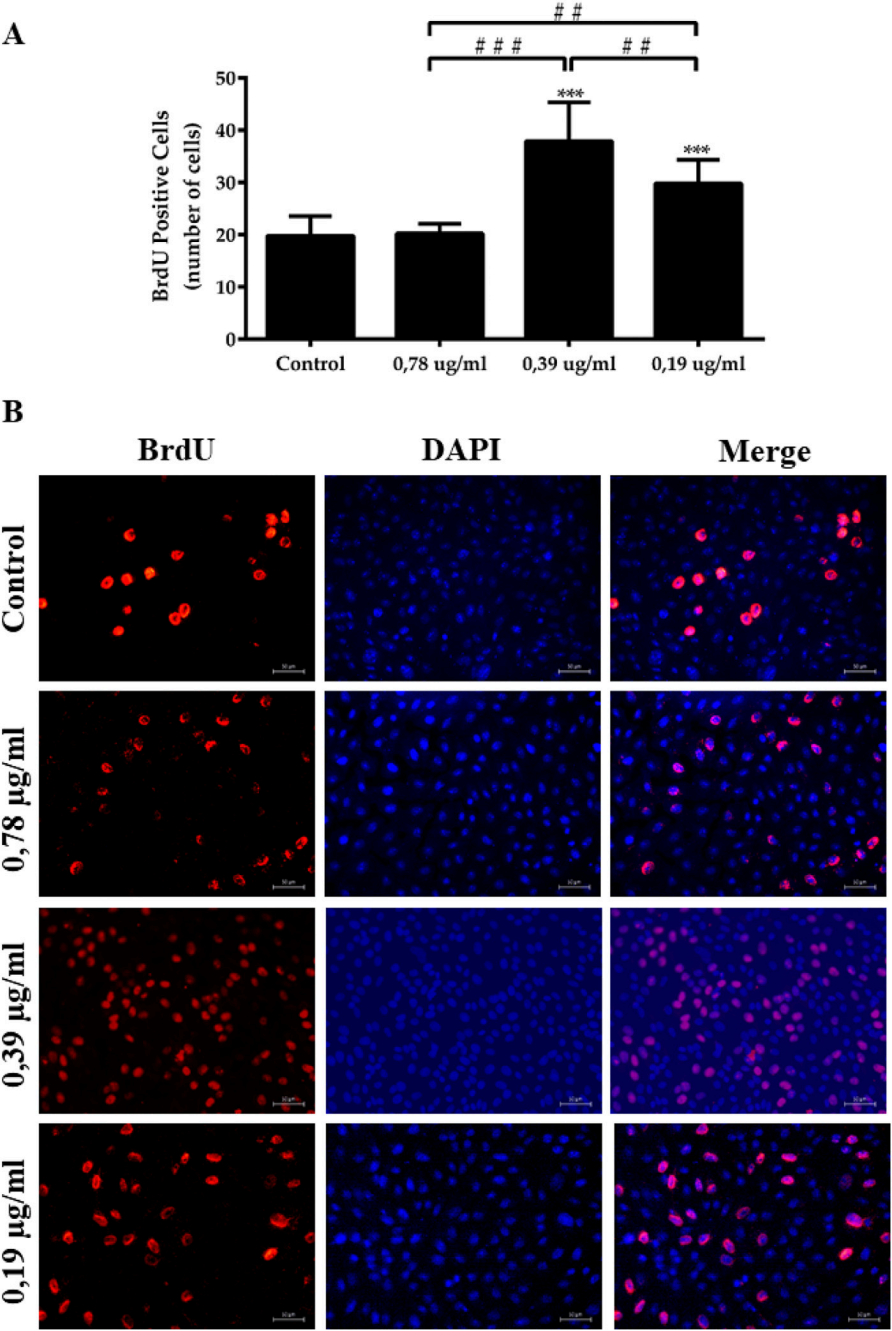
Cell proliferation induced by the ESML extract. Cells incorporated with BrdU and stained with anti-BrdU antibodies, the control group and ESML groups 0.78 μg/mL, 0.39 μg/mL and 0.19 μg/mL in 24 h were analyzed. **(A)** Quantitative data **(B)** Immunofluorescence macrophotography. Significance was considered at *P < 0.05, ***P < 0.001 vs control; ^##^P < 0.01 vs 0.39 μg/mL and 0.78 μg/mL, ^###^P < 0.001 vs 0.78 μg/mL. It was performed in triplicate. Mean ± standard deviation. Scale bar in microphotography = 50 µm.

The concentrations of 0.39, and 0.19 μg/mL of ESML significantly increased the proliferation of fibronblasts compared to the control group, with 37.89 ± 7.41 and 29.78 ± 4.52 BrdU positive cells, respectively (Figure 9).

Accordingly, the group of cells exposed to the ESML extract at concentrations of 0.39 μg/mL and 0.19 μg/mL showed increased cell proliferation, with a higher number of BrdU-positive cells compared to the control group. The 0.78 μg/mL concentration of the ESML extract did not significantly promote cell proliferation compared to the control group over 24 h.

**FIGURE 10:**
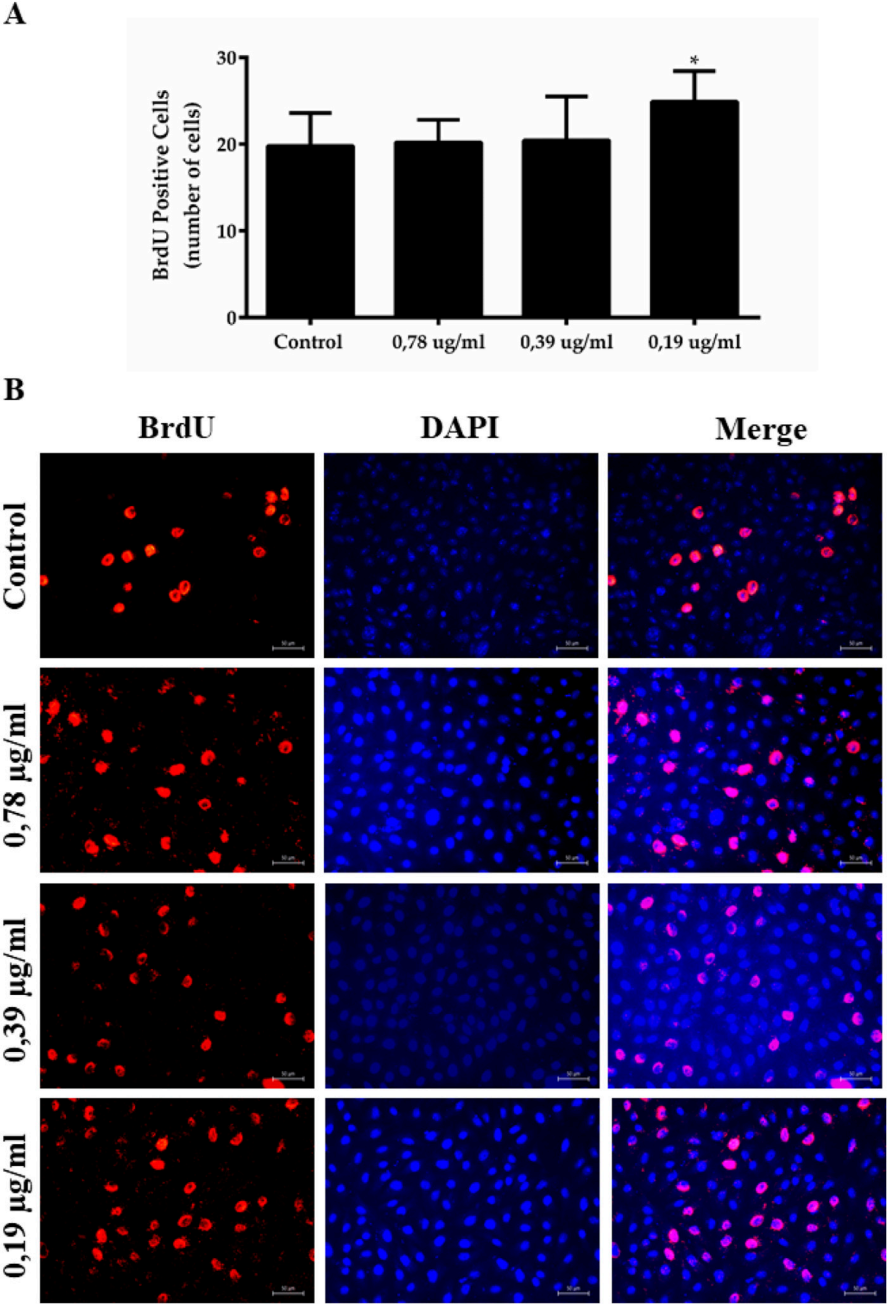
Cell proliferation induced by the EPML extract. Cells incorporated with BrdU and stained with anti-BrdU antibodies, the control group and EPML groups 0.78 μg/mL, 0.39 μg/mL and 0.19 μg/mL in 24 h were analyzed. **(A)** Quantitative data, **(B)** Immunofluorescence macrophotography. Significance was considered at *P < 0.05, ***P < 0.001 vs control; ^##^P < 0.01 vs 0.39 μg/mL and 0.78 μg/mL, ^###^P < 0.001 vs 0.78 μg/mL. It was performed in triplicate. Mean ± standard deviation. Scale bar in microphotography = 50 µm.

The concentration of 0.19 μg/mL of EPML also significantly induced the proliferation of L929 cells compared to the control, showing 24.89 ± 3.55 BrdU positive cells (Figure 10).

Thus, the EPML extract at a concentration of 0.19 μg/mL, after 24 h in contact with the cells, promoted increased proliferation compared to the control group, resulting in a higher number of BrdU positive cells in the group exposed to the EPML extract (0.19 μg/mL).

## 4 Discussion

The botanical drugs and their metabolites have been used throughout human history for the treatment of wounds (Shedoeva et al., 2019). In this study, we demonstrated the wound healing activity of *M. linifera in vitro*. We observed the presence of metabolites from the classes of terpenes, saponins, and phenolic compounds in ECML and EPML, according to the results obtained through HPTLC. These metabolites are widely found in numerous botanical species and possess various pharmacological activities, including wound healing (Kim et al., 2011; Nadaf et al., 2018; Saoudi et al., 2021).

The study by Faria et al. (2021) demonstrates that the stem extract of *M. linifera* contains tannins and polyphenols, as well as saponins. It was also demonstrated by Trujillo-Trujillo et al. (2006) that the stem extract of *M. linifera* contains metabolites such as polyphenols and saponins, thereby corroborating our findings regarding the characterization of the extracts.

In the analysis performed using RP-UHPLC-HRMS, seven major substances were detected in ESML and eleven in EPML, with five substances identified in both extracts (gallic acid, pinellic acid, suberic acid, coriolic acid, and p-coumaric acid). Among these substances, gallic acid and p-coumaric acid are phenolic compounds, which is consistent with the results of the HPTLC assay. Not all of the 13 metabolites identified in the two extracts studied in this work share the same chemical composition. The RP-UHPLC-HRMS analysis detected the following: phenolic compounds - non-flavonoids (p-coumaric acid, 4-hydroxycinnamic acid methyl ester), flavonoids (gallic acid, schaftoside isomers, caffeic acid, quercetin, rutin, isorhamnetin), fatty acids (pinellic acid, suberic acid, coriolic acid), disaccharides (sucrose), and alkaloids (isoschaftoside isomers).

The HPTLC assay did not reveal bands corresponding to flavonoids and alkaloids, whereas the RP-UHPLC-HRMS assay detected metabolites from these classes of chemical. This discrepancy is likely due to the greater sensitivity, specificity, and accuracy of RP-UHPLC-HRMS compared to HPTLC (Ma et al., 2022). However, this does not invalidate the data obtained from the HPTLC bands.

Among the metabolites identified in the extracts is gallic acid, a flavonoid found in various botanical species, which exhibits multiple bioactivities such as antioxidant, anti-inflammatory, antitumoral, and wound-healing properties (BenSaad et al., 2017; Choi et al., 2009; Zhang et al., 2019).

It has been demonstrated that gallic acid promotes the migration of fibroblasts and keratinocytes cultured under both normal and hyperglycemic conditions, in addition to enhancing the expression of proteins such as focal adhesion kinase (FAK), c-Jun N-terminal kinases (JNK), and extracellular signal-regulated kinases (Erk). This suggests that this metabolite contributes to improved wound healing *in vitro* (Yang et al., 2016). These findings support our results showing enhanced fibroblast migration at the lowest concentrations tested of ESML and EPML, as the extracts also contain gallic acid.

Another metabolite identified in the ESML and EPML extracts is suberic acid, which, in addition to being found in various plant species, can also be endogenously produced by humans (Pettersen et al., 1972) In a previous study, it was demonstrated that suberic acid could act as a natural ligand for olfactory receptors 10 subfamily A member 3 (OR10A3). This receptor, besides being expressed in olfactory sensory neurons, is also present in human dermal fibroblasts. The interaction of this metabolite with fibroblasts activates signaling pathways such as cAMP/Akt, which may potentially benefit the wound healing process. However, the experimental model tested could not confirm this hypothesis (Kang et al., 2022).

In our murine fibroblast model, suberic acid may also interact with certain members of the olfactory receptor (OR) family. However, it is not possible to assert that the specific OR family member modulated in mice is the same as that in human cells.

The p-Coumaric acid is a phenolic acid identified in the ESML and EPML extracts in our analysis. A study demonstrated that the aqueous extract of *Myrciaria plinioides* exhibits *in vitro* wound-healing activity, attributed to the presence of p-coumaric acid and/or phenolic glycosides. In the same study, isolated p-coumaric acid, caffeic acid, and quercetin were tested, revealing that quercetin stimulated cell migration compared to the control, while p-coumaric acid induced both migration and proliferation of L929 cells, possibly associated with increased TGF-β release (Marmitt et al., 2024) Thus, p-coumaric acid exhibits *in vitro* wound-healing activity, which may contribute to making ESML and EPML extracts strong candidates for wound-healing applications.

During the injury process, there is an excessive production of reactive oxygen species (ROS), which leads to the progression of skin lesions through oxidative stress and delays wound healing (Wang et al., 2023; Gonçalves et al., 2022). Therefore, ESML and EPML may contribute to the elimination of excess ROS during wound healing.

The serial concentration curves of ESML and EPML in the cytotoxicity test demonstrated that at the lower concentrations, ESML did not exhibit cellular toxicity. However, after 24 h of cell exposure to ESML at the lower concentrations (3.125 μg/mL to 0.19 μg/mL), there was a significant increase in MTT metabolism and formazan crystal formation compared to the control, thereby increasing cell viability rates. At 48 and 72 h, the same effect was observed at the lowest concentration of ESML (0.19 μg/mL). EPML was also non-cytotoxic and showed an increase in viability only at the lowest concentration (0.19 μg/mL) after 48 h of exposure. This likely occurred due to increased fibroblast proliferation induced by the extracts at the lower concentrations tested, especially by ESML.

The increased viability of L929 cells treated with p-coumaric acid or caffeic acid individually has already been described, supporting our findings, as these metabolites were identified predominantly in the ESML and EPML extracts (Marmitt et al., 2024). Based on these findings in the cytotoxicity test, the three lowest concentrations (0.78 μg/mL, 0.39 μg/mL, and 0.19 μg/mL) were used for further assays.

When a scratch assay was performed on fibroblast monolayers and subsequently treated with ESML, there was a significant increase in cell migration after 24 h at concentrations of 0.39 μg/mL, and 0.19 μg/mL, whereas EPML induced a significant increase in cell migration only at a concentration of 0.19 μg/mL after 24 h, thereby significantly reducing the wound area in these groups. Some studies have demonstrated that the modulation of cytoskeletal and adhesion protein expression is crucial for promoting cell migration and motility (Cen et al., 2022; Tang et al., 2020; Kuwahara et al., 2001).

BrdU immunolabeling was performed to demonstrate the increase in cell proliferation induced by the *M. linifera* extracts, as suggested by the results observed in the MTT assay. In the proliferation test, there was a significant increase in the number of BrdU positive cells after 24 h of exposure to 0.39 μg/mL, 0.19 μg/mL of ESML, and 0.19 μg/mL of EPML. Thus, ESML and EPML modulated the cell cycle of fibroblasts, enhancing their mitotic activity. Cell migration and proliferation are important events during the tissue repair process, occurring during the proliferation phase of wound healing (Gurtner et al., 2008).

*In vivo*, the migration and proliferation of fibroblasts from the tissue adjacent to the injury are necessary for their differentiation into myofibroblasts, enabling them to exert contractile force for wound edge contraction and closure. Furthermore, fibroblasts contribute to the formation of the extracellular matrix through the synthesis of extracellular matrix proteins, such as collagen, thereby forming granulation tissue (Li and Wang, 2011; Chen et al., 2017).

The presence of p-coumaric acid in the ESML and EPML extracts may be modulating the process of cell proliferation, as it was observed that fibroblasts treated with p-coumaric acid and the cell proliferation inhibitor mitomycin C did not exhibit increased cell migration *in vitro* when compared to the group treated with p-coumaric acid alone. Thus, the wound closure observed *in vitro* in the referenced study occurred due to cell proliferation. Furthermore, it was demonstrated that this metabolite modulated the cell cycle (Aquino et al., 2021).

A study by Csepregi et al. (2020) demonstrated the antioxidant and wound-healing activities of phenolic compounds present in certain medicinal plants (Csepregi et al., 2020). Natural products, such as olive oil, contain phenolic compounds, which have been shown to promote the healing of skin wounds by stimulating fibroblasts in culture (Melguizo-Rodríguez et al., 2021). Thus, the *in vitro* wound-healing activity of ESML and EPML may be attributed to the presence of phenolic compounds.

Furthermore, it has been demonstrated that phenolic content of botanical origin increases collagen levels in lesions and accelerates the epithelialization of wound matrix in diabetic rats (Kaltalioglu et al., 2020). These processes are fundamental for wound healing progression and lesion closure.

In light of the above, we demonstrated *in vitro* the wound healing potential of ESML and EPML; these extracts modulate the cell cycle, increasing fibroblast proliferation, and may also modulate the expression of proteins responsible for cell migration. This is a pioneering study investigating the wound healing effects of ESML, and EPML. Although we have shown that ESML, and EPML have wound healing potential is necessary to conduct biomonitoring to investigate whether the wound-healing activity of both extracts occurs due to the predominant effect of one of the thirteen metabolites identified in our findings or through their synergistic effects. It is also important to continue the *in vitro* study with other types of cells that play a crucial role in the wound-healing process, such as keratinocytes, and macrophages. *In vivo* studies are also needed, in addition to performing assays to verify the phase of healing that is being modulated by ESML and EPML and their mechanisms of action, for example, inflammatory markers assay activity and collagen synthesis.

However, the results obtained in this study are encouraging, as our research is the first to demonstrate that *M. linifera* possesses wound healing properties, with its metabolites being involved in the processes of cell migration and proliferation. This finding scientifically validates the ethnopharmacological practice that has been used for decades by the Amazonian population (Plowman, 1969).

As we advance this study to elucidate the mechanisms of action and modulation pathways of wound healing induced by the metabolites of these extracts, as previously described, it will be possible to leverage modern technologies in combination with this botanical drug. This approach may enable the development of formulations, carrier-based dressings, and other therapeutic strategies for the efficient treatment of chronic wounds.

## Data Availability

The original contributions presented in the study are included in the article/Supplementary Material, further inquiries can be directed to the corresponding author.
