# Supplementary material for: Montrichardia linifera (Arruda) Schott promotes accelerated wound healing in vitro: a promising healing
Source: Front Pharmacol. 2025 May 20;16:1512570. doi: 10.3389/fphar.2025.1512570 (PMC12130024; doi:10.3389/fphar.2025.1512570)
Supplement: Supplementary file 1 [file DataSheet1.docx]

Supplementary Material

# Supplementary Figures

**Supplementary Figure 1.** Viability of L929 cells exposed for 72 hours to different concentrations of DMSO. Significance was considered at *P < 0.05, **P < 0.01, ***P < 0.001 vs. control. All groups were used n = 8, performed in triplicate. Mean ± standard deviation.
